# Low Plasma Sphingomyelin Levels Show a Weak Association with Poor Neurological Outcome in Cardiac Arrest Patients: Results from the Prospective, Observational COMMUNICATE Trial

**DOI:** 10.3390/jcm9040897

**Published:** 2020-03-25

**Authors:** Aurelio Boerlin, Tanja Luescher, Christoph Becker, Sebastian Perrig, Emanuel Thommen, Madlaina Widmer, Katharina Beck, Alessia Vincent, Kai Tisljar, Luca Bernasconi, Peter Neyer, Philipp Schuetz, Raoul Sutter, Stephan Marsch, Sabina Hunziker

**Affiliations:** 1 Intensive Care Unit, University Hospital Basel, University of Basel, 4031 Basel, Switzerland; aurelio.boerlin@gmail.com (A.B.); tanja.luescher@vtxmail.ch (T.L.); kai.tisljar@usb.ch (K.T.); raoul.sutter@usb.ch (R.S.); stephan.marsch@usb.ch (S.M.); 2 Medical Communication and Psychosomatic Medicine, University Hospital Basel, 4031 Basel, Switzerland; christoph.becker@usb.ch (C.B.); sebastian.perrig@gmail.com (S.P.); ebthommen@gmx.net (E.T.); madlainawidmer@gmail.com (M.W.); juliakatharina.beck@usb.ch (K.B.); alessiamichelle.vincent@usb.ch (A.V.); 3 Institute of Laboratory Medicine, Kantonsspital Aarau, 5001 Aarau, Switzerland; luca.bernasconi@ksa.ch (L.B.); peter.neyer@ksa.ch (P.N.); 4 Medical faculty of the University of Basel, 4001 Basel, Switzerland; philipp.schuetz@ksa.ch; 5 Department of Internal Medicine, Kantonsspital Aarau, 5001 Aarau, Switzerland; 6 Department of Neurology, University Hospital Basel, 4031 Basel, Switzerland

**Keywords:** sphingomyelin, cardiac arrest, neurological outcome

## Abstract

There is interest in novel blood markers to improve risk stratification in patients presenting with cardiac arrest. We assessed associations of different plasma sphingomyelin concentrations and neurological outcome in patients with cardiac arrest. In this prospective observational study, adult patients with cardiac arrest were included upon admission to the intensive care unit (ICU). We studied associations of admission plasma levels of 15 different sphingomyelin species with neurological outcome at hospital discharge (primary endpoint) defined by the modified Rankin Scale by the calculation of univariable and multivariable logistic regression models adjusted for age, gender, and clinical shock markers. We included 290 patients (72% males, median age 65 years) with 162 (56%) having poor neurological outcome at hospital discharge. The three sphingomyelin species SM C24:0, SM(OH) C22:1, and SM(OH) C24:1 were significantly lower in patients with poor neurological outcome compared to patients with favorable outcome with areas under the curve (AUC) of 0.58, 0.59, and 0.59. SM(OH) C24:1 was independently associated with poor neurological outcome in a fully-adjusted regression model (adjusted odds ratio per log-transformed unit increase in SM(OH) C24:1 blood level 0.18, 95% CI 0.04 to 0.87, *p* = 0.033). Results were similar for 1-year mortality. Low admission sphingomyelin levels showed a weak association with poor neurological outcome in patients after cardiac arrest. If validated in future studies, a better understanding of biological sphingomyelin function during cardiac arrest may help to further advance the therapeutic approach and risk stratification in this vulnerable patient group.

## 1. Introduction

Cardiac arrest remains a severe condition with high mortality and morbidity [1]. Among survivors, risk for irreversible brain injury is high, leading to severe disability [2]. The discussion of therapeutic options in this context is often challenging, since patients are usually unconscious upon arrival to the intensive care unit (ICU), and isolated prognostic markers are not reliable. However, reliable and accurate prognostic information is crucial to guide objective and informed decision-making respecting the patient’s presumed will [3]. Research investigating prognostic blood markers in this patient group has the potential to improve the prediction of clinical outcomes and may help to define new therapeutic opportunities [4,5]. 

In the field of metabolomics—the analysis of a subset of metabolites under a defined clinical condition [6,7]—sphingolipids (SLs) have been linked to atherosclerosis, coronary artery disease (CAD), Alzheimer’s disease, and sepsis [8,9,10,11,12,13,14,15,16]. SLs represent a class of lipids that are highly concentrated in the membranes of eukaryotes [17]. Three main groups of sphingolipids can be differentiated: ceramides, which are bioactive molecules involved in cellular proliferation, growth, and apoptosis, and their metabolites sphingomyelin (SM) and glycosphingolipids [17,18]. Particularly, SM is highly expressed in the central nervous system (CNS), peripheral nerve tissue, ocular lenses, and erythrocytes [19]. Its levels are of central importance in modulating structural plasma membrane (PM) properties and mediating cell signals [18]. The regulation of SM content in a cell is directed by two main groups of enzymes: sphingomyelin synthases (SMSs), which catalyse the SM synthesis from ceramides and sphingomyelinases (SMases), which are responsible for SM hydrolysis to generate ceramides and phosphocholine [18]. 

In patients with sepsis, ceramide/SM ratios were consistently elevated due to the activation of SMases and acted as significant discriminators between surviving and non-surviving patients [8]. A similar effect was found in an in vitro study, where, during cerebral ischemia, SM concentrations dropped and ceramide concentrations rose in cells of the cerebral cortex [18]. Higher plasma SM values compared to healthy controls have been linked to subclinical atherosclerosis [13] and coronary artery disease [10,14]. However, they did not show a significant association with incident coronary artery disease in adults free of cardiovascular disease [16]. In patients undergoing coronary angiography, four types of SM showed a positive association with mortality, while five SM groups had a protective effect [15]. This finding indicates that individual species of SM may have different roles. 

To our knowledge, no study has yet examined the potential of SMs in patients with cardiac arrest. Since the ceramide–SM pathway has been shown to be involved in the pathophysiology of sepsis and coronary artery disease, we were interested in evaluating plasma SM levels in a well-characterized cohort of patients with cardiac arrest regarding neurological outcome at hospital discharge and all-cause mortality within 1 year after cardiac arrest.

## 2. Experimental Section

This is a preplanned analysis of patients included in an ongoing prospective observational study (COMMUNICATE trial) at the University Hospital of Basel in Switzerland. The COMMUNICATE trial collects clinical and laboratory data of patients with cardiac arrest. It was approved by the Ethics Committee of Northwest and Central Switzerland (Ethikkommission Nordwest und Zentralschweiz, EKNZ) and complies with the Declaration of Helsinki. Patients or, in case of unconsciousness, a relative of theirs or a health care agent had to sign a consent form to participate in the study. Detailed information about the study has been published elsewhere [5,20,21,22].

The overall hypothesis of this study is that admission levels of different sphingomyelin species are associated with poor neurological outcome at hospital discharge and mortality at discharge and within 1 year in patients with out-of-hospital cardiac arrest (OHCA).

Between October 2012 and June 2018, we enrolled patients after cardiac arrest upon intensive care unit (ICU) admission. There were no exclusions regarding patient characteristics and type, severity, or duration of cardiac arrest. Family members and, if the medical situation allowed, patients were informed about the study and were asked for informed consent. Exclusion criteria were monitored in-hospital cardiac arrest (IHCA), lack of consent, or lack of blood sampling for later measurements of sphingomyelin.

Upon admission to the ICU, blood samples from each patient were drawn for routine chemistry measurements and additionally, serum samples were aliquoted and frozen at −80 °C until retesting for the measurement of metabolomics biomarkers [23,24,25,26]. As secondary tubes, conical false bottom tubes made of polyethylene with a lamellar plug were used. Laboratory testing was done using the AbsoluteIDQ p180 kit and analyzed using MetIDQ™ software (Biocrates Life Sciences AG, Innsbruck, Austria). Serum SM levels were quantified using flow injection tandem mass spectrometry analysis (FIA-MS/MS) at Biocrates Life Sciences (Innsbruck, Austria) [27]. Samples were prepared according to the manufacturer’s protocol. Fifteen unique SM species were measured, differing in the length of their fatty acid chain (SM C16:0, SM C16:1, SM C18:0, SM C18:1, SM C20:2, SM C22:3, SM C24:0, SM C24:1, SM C26:0, SM C26:1 as well as hydroxylated species SM(OH) C14:1, SM(OH) C16:1, SM(OH) C22:1, SM(OH) C22:2, SM(OH) C24:1). Clinical parameters on arrival such as blood pressure and heart rate were collected as well as initial cardiac arrest parameters (i.e., no-flow time (time from cardiac arrest to start of basic life support (BLS)), low-flow time (time from start of BLS to return of spontaneous circulation (ROSC)), cardiac arrest setting, bystander observing the cardiac arrest and providing cardiopulmonary resuscitation (CPR), initial rhythm), sociodemographics (i.e., age, gender, smoking status) and comorbidities (i.e., coronary artery disease, congestive heart failure, hypertension, diabetes, hyperlipidemia, liver failure, and renal failure). The cause of cardiac arrest was determined by chart review relying on the information from the treating medical team.

The primary endpoint was defined as neurological outcome at hospital discharge from the primary facility, which was defined by the modified Rankin Scale (mRS), which consists of 7 levels. Levels 0 (no symptoms), 1 (no significant disability despite symptoms; able to carry out all usual duties and activities), and 2 (slight disability; unable to carry out all previous activities, but able to look after own affairs without assistance) were classified as favorable outcomes, whereas levels 3 (moderate disability; requiring some help, but able to walk without assistance), 4 (moderately severe disability; unable to walk and attend to bodily needs without assistance), 5 (severe disability; bedridden, incontinent, and requiring constant nursing care and attention), and 6 (dead) were defined as poor outcomes [28,29]. 

Secondary outcomes were defined as in-hospital mortality and long-term all-cause mortality at 1 year after cardiac arrest.

To characterize the patient cohort, descriptive statistics including medians and inter-quartile ranges were used for continuous variables as appropriate. Frequencies were reported for binary or categorical variables. We used Spearman-rank tests to investigate the correlations of SM species and predefined variables, including initial vital signs (i.e., heart rate, respiratory rate, systolic and diastolic blood pressure), resuscitation measures (i.e., no-flow and low-flow time), and routine blood markers (i.e., troponin, creatinine, urea, lactate, and pH). Univariate and multivariate logistic regression models were calculated to evaluate the association of SM levels with the primary and secondary endpoints. To achieve a normal distribution, data of SM levels were log transformed with a base of 10. Odds ratios (OR) and 95% confidence intervals (CI) were reported as a measure of association. Covariates used in the multivariate analyses were defined prior to testing. Three multivariate models were calculated, adjusted for age and gender (A), age, gender, and comorbidities (B), and finally a model adjusted for age, gender, and clinical markers of shock (heart frequency, respiratory frequency, systolic and diastolic blood pressure (SBP, DBP), temperature, Glasgow Coma Scale (GCS)) (C). To assess discrimination, we calculated the area under curve (AUC). STATA 12.0 and STATA 15.0 were used for all statistical analyses, and a two-sided *p*-value of <0.05 was considered significant.

## 3. Results

### 3.1. Baseline Characteristics

From October 2012 until June 2018, 406 adult patients were admitted to the ICU at the University hospital of Basel following cardiac arrest, of which 290 (71.4%) had an admission blood sample available and were included in this study. Baseline characteristics stratified by neurological outcome according to modified Rankin Scale (mRS) at hospital discharge are shown in Table 1. Out of 290 patients, 162 (55.9%) had a poor neurological outcome. Patients with poor neurological outcome had lower SM levels in 3 out of 15 analyzed species, namely in SM C24:0, SM(OH) C22:1, and SM(OH) C:24:1 (Table 2).

### 3.2. Spearman Rank Correlation

We evaluated the correlation between SM species and the predefined clinical variables, resuscitation parameters, and laboratory shock markers (Table 3). We found correlations between diastolic blood pressure and all SM species. Additionally, there was a significant correlation between SM C16:1, SM C18:1, and SM C 22:3 and initial pH. Finally, SM C24:0 showed a significant correlation with initial troponin, creatinine, and urea.

### 3.3. Primary Outcome

**Figure 1:**
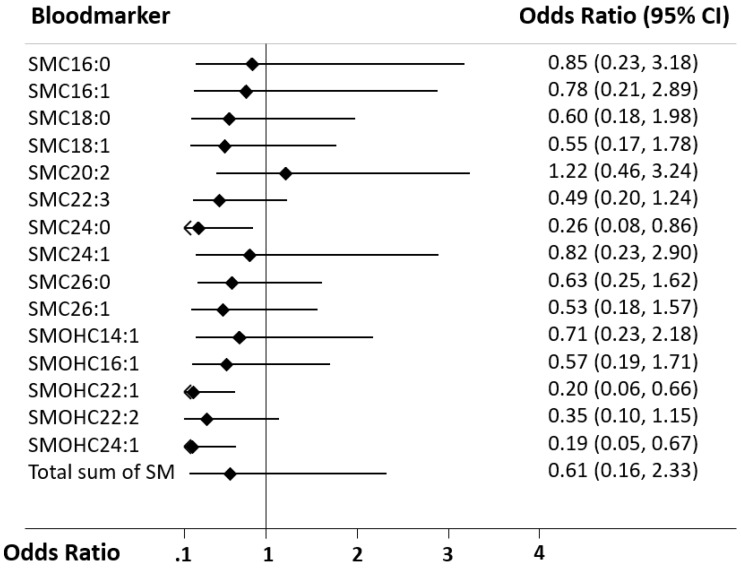
Forest plot for SM markers in regard to the primary endpoint (neurological outcome). Data is presented as odds ratio (OR) and 95% confidence interval (95% CI). SM: Sphingomyelin.

**Figure 2:**
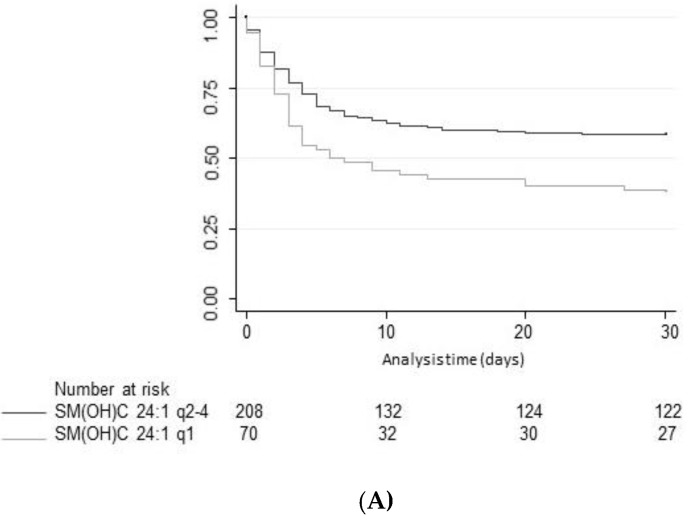

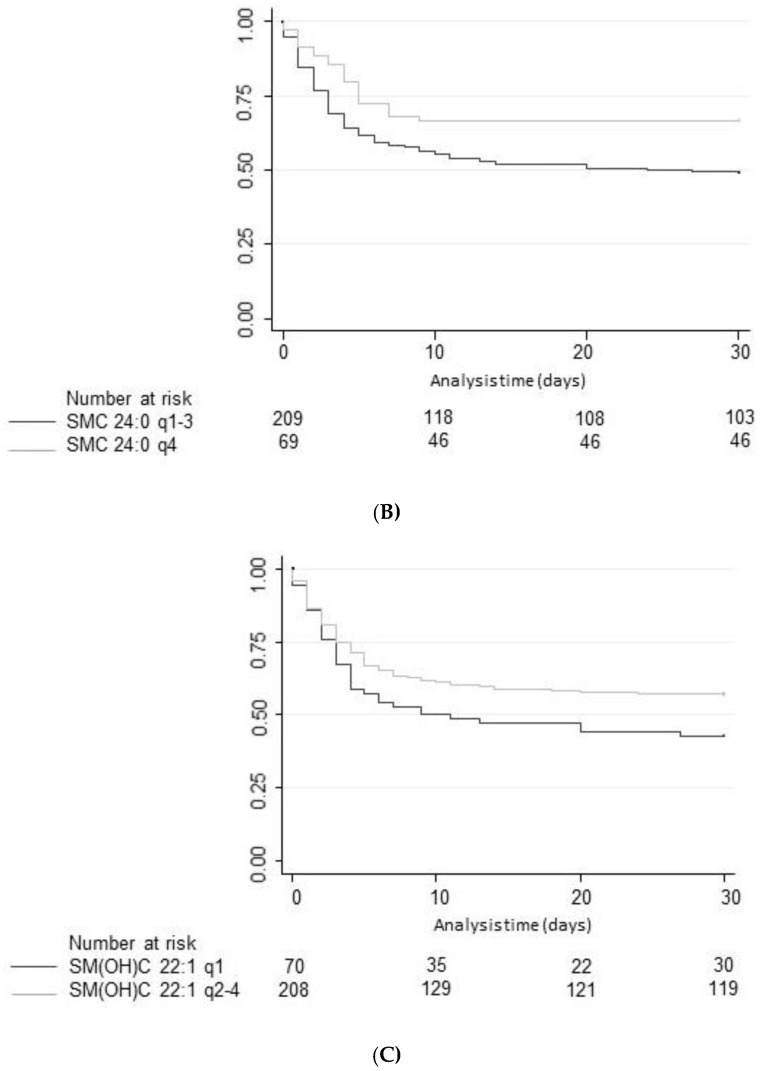
(**A**) Kaplan–Meier survival estimates at 30-day assessment looking at SM(OH)C 24:1. (**B**) Kaplan-Meier survival estimates at 30-day assessment looking at SMC 24:0. (**C**) Kaplan-Meier survival estimates at 30-day assessment looking at SM(OH)C 22:1. Data is presented as number (*n*) and percentage (%). SM: Sphingomyelin; q: Quartile.

First, we calculated a univariate logistic model to investigate associations between SM species and neurological outcome at discharge, which was our primary endpoint (Table 2 and Figure 1). SM C24:0 (OR 0.26, 95%CI 0.08 to 0.86), SM(OH) C22:1 (OR 0.2, 95%CI 0.06 to 0.66) and SM(OH) C24:1 (OR 0.19, 95%CI 0.05 to 0.67) were significantly associated with neurological outcome in univariate analyses. The respective AUCs were 0.58, 0.59, and 0.59. Figure 2 shows Kaplan–Meier analysis of the three significant SM species and mortality. These metabolites were also significantly associated in a multivariate analysis adjusted for age and gender, with the strongest associations found for SM(OH) C24:1 (OR 0.18, 95%CI 0.05 to 0.68). In a fully adjusted model additionally adjusted for comorbidities and shock parameters, only SM(OH) C24:1 remained significantly associated with poor neurological outcome with an adjusted odds ratio of 0.18 (95%CI 0.04 to 0.87, *p* = 0.033) (Table 2 and Table A2).

In an additional sensitivity analysis, we included primary rhythm and low-flow time into the model and found similar results regarding the association with the primary endpoint.

### 3.4. Secondary Outcomes

A total of 161 participants (55.5%) survived the initial stay at the University hospital and were discharged alive. After 1 year, 120 participants (41.4%) were still alive. In univariate analysis, SM C24:0, SM(OH) C22:1, and SM(OH) C24:1 were again significantly associated with in-hospital mortality and all-cause mortality after 1 year (Table A1 and Table A4). The respective AUCs were 0.56, 0.57, and 0.57 for in-hospital mortality and 0.57, 0.59, and 0.58 for all-cause mortality after 1 year. However, in the multivariate analysis, there was no significant association of the different metabolites and mortality (Table A3 and Table A5).

## 4. Discussion

Improving the pathophysiological understanding of outcomes and early risk stratification in cardiac arrest patients has recently become a research priority with high potential to improve the management of these patients. Still, current risk scores and blood markers show a suboptimal performance regarding outcome of patients and a “bundle approach” including several markers and clinical parameters from distinct pathophysiological pathways may be needed to further advance the field. Therefore, the evaluation of novel potential marker candidates from metabolic pathways is an interesting approach for research. Herein, the present study assessed the value of 15 different SM species to predict neurological outcome according to mRS and all-cause mortality until hospital discharge and within 1 year of admission. In univariate and multivariate models, high plasma levels of three SM metabolites, namely SM C24:0, SM(OH) C22:1, and SM(OH) C24:1 were found to be associated with better neurological outcome. However, discrimination was only moderate and lower than other established brain damage markers such as neuron-specific enolase (NSE) [21]. For mortality, univariate associations were also found, but none of the markers was independently associated with outcome, and all markers had poor performance when looking at discrimination. 

We found heterogeneity regarding the 15 different SM species and outcome prediction with only 3 showing significant results in univariate analysis. In addition, the sum of all SM species did not show strong associations with neurological outcome or mortality. These results are in line with a large, prospective, multicenter trial, where Yeboah et al. described that total SM levels showed no significant associations with incident coronary heart disease event rates [16]. Thus, rather than the total amount of SM, single metabolites seem to play a more important role than the total amount of SM, and better understanding the specific functions of these SM proteins may help to improve our pathophysiologic understanding of diseases. 

Furthermore, Drobnik et al. showed that a higher ceramide/SM ratio predicted mortality in patients with a septic shock [8]. A similar finding was published by Ferrario et al., where SM C20:2 was decreased in non-survivors of a septic shock [9]. We did not measure ceramides in our sample and can thus not replicate their findings. Hence, further research is needed to examine ceramides in conjunction with SM levels in patients after OHCA to understand if this would yield better results. 

The strength of this study includes the measurement and analysis of different SM species to examine their predictive power separately. Furthermore, we examined the values of plasma SM in a unique patient group, in which SM levels have not been examined before. 

We are aware of the following limitations of our study. First, no measurements of ceramides, another class of SL showing predictive power in patients with sepsis [8], were conducted due to a lack of additional specimens. Yet, SM levels are dependent on de novo synthesis from ceramide catalyzed by the enzymes SMases. In addition, ceramide/sphingomyelin ratios could have been calculated, which has also shown to be an interesting computation for outcome [8,10]. Therefore, a measurement of ceramides or SMases would have provided further insight into the SM pathway in patients with cardiac arrest. Additionally, the time interval between cardiac arrest and collection of SM measurements has not been recorded. This measurement would have been valuable to study the dynamics over time of SM levels in our patient group. 

## 5. Conclusions

Low admission sphingomyelin levels showed a weak association with poor neurological outcome in patients after cardiac arrest. If validated in future studies, a better understanding of biological sphingomyelin function during cardiac arrest may help to further advance the therapeutic approach and risk stratification in this vulnerable patient group.

## Figures and Tables

**Table 1:** Baseline characteristics.

*n*, %	All	mRS 0–2	mRS 3–6	*p*-Value
	290	128 (44.1%)	162 (55.9%)	
**Sociodemographics**				
**Age years, median (IQR)**	64.7 (56.5, 74.1)	62.5 (52.5, 72.6)	67.9 (57.8, 77.8)	0.005
**Male gender, *n* (%)**	210 (72.4%)	108 (84.4%)	102 (63.0%)	<0.001
**Comorbidities**				
**Coronary artery disease, *n* (%)**	196 (67.8%)	102 (80.3%)	94 (58.0%)	<0.001
**Congestive heart failure, *n* (%)**	42 (14.5%)	18 (14.2%)	24 (14.8%)	0.88
**COPD, *n* (%)**	21 (7.3%)	5 (3.9%)	16 (9.9%)	0.054
**Liver disease, *n* (%)**	5 (1.7%)	0 (0.0%)	5 (3.1%)	0.046
**Hypertension, *n* (%)**	152 (52.6%)	70 (55.1%)	82 (50.6%)	0.45
**Diabetes, *n* (%)**	69 (23.9%)	22 (17.3%)	47 (29.0%)	0.021
**Chronic renal failure, *n* (%)**	38 (13.1%)	16 (12.6%)	22 (13.6%)	0.81
**Malignant disease, *n* (%)**	30 (10.4%)	6 (4.7%)	24 (14.8%)	0.005
**Neurological disease, *n* (%)**	31 (10.7%)	8 (6.3%)	23 (14.2%)	0.031
**Smoking status**				
**Never, *n* (%)**	93 (38.9%)	39 (34.2%)	54 (43.2%)	0.34
**Smoker, *n* (%)**	74 (31.0%)	39 (34.2%)	35 (28.0%)	0.28
**Ex-smoker, *n* (%)**	72 (30.1%)	36 (31.6%)	36 (28.8%)	0.67
**Resuscitation Circumstances**				
**No-flow time (min), median (IQR)**	0 (0, 7)	0 (0, 2)	3 (0, 10)	<0.001
**Low-flow time (min), median (IQR)**	15 (10, 26)	12 (7, 20)	20 (13, 30)	<0.001
**Bystander CPR, *n* (%)**	190 (65.7%)	104 (81.2%)	86 (53.4%)	<0.001
**Shockable initial rhythm, *n* (%)**	170 (58.8%)	102 (79.7%)	68 (42.2%)	<0.001
**Initial status, ICU**				
**Systolic blood pressure (mmHG) day 0, median (IQR)**	117 (101, 130)	120 (104, 133)	114 (97, 129)	0.063
**Diastolic blood pressure (mmHG) day 0, median (IQR)**	66 (55, 77)	70 (59, 79)	63.5 (51, 76)	0.007
**Heart rate (bpm) day 0, median (IQR)**	85 (74, 99)	81 (70, 94)	89 (76, 103)	0.002
**Respiratory rate (/min)—day 0, median (IQR)**	16 (14, 20)	17 (14, 19)	16 (14, 20)	0.39
**Temperature (degree Celsius) day 0, median (IQR)**	35.7 (34.9, 36.3)	36 (35.4, 36.5)	35.5 (34.6, 36.1)	<0.001
**GCS day 0, median (IQR)**	3 (3, 5)	5 (3, 15)	3 (3, 3)	<0.001
**Blood markers**				
**Initial pH day 0, median (IQR)**	7.26 (7.17, 7.33)	7.3 (7.24, 7.34)	7.23 (7.11, 7.31)	<0.001
**Initial lactate (mmol/L) day 0, median (IQR)**	6.1 (3.6, 9)	4.55 (2.7, 6.35)	7.6 (5.2, 10.1)	<0.001
**Creatinine (umol/L) day 0, median (IQR)**	95 (78, 119)	88 (77, 108)	102 (80, 132)	0.003
**Urea (mmol/L) day 0, median (IQR)**	7.2 (5.6, 9.1)	6.7 (5.4, 8.35)	7.5 (6, 10.3)	0.023
**Cause of cardiac arrest**				
**Coronary artery disease, *n* (%)**	141 (48.8%)	75 (59.1%)	66 (40.7%)	0.002
**Initial arrhythmia, *n* (%)**	57 (19.7%)	29 (22.8%)	28 (17.3%)	0.24
**Respiratory, *n* (%)**	91 (31.5%)	23 (18.1%)	68 (42.0%)	<0.001

Data are presented as median (interquartile range) or number (*n*) and percentage (%). COPD: Chronic obstructive pulmonary disease, IQR: inter-quartile range, mRS: modified Rankin Scale, GCS: Glasgow Coma Scale.

**Table 2:** Univariate and multivariate analysis for modified Rankin Scale (mRS) at hospital discharge.

	mRS 0–2, Median (IQR), mg/dL	mRS 3–6, Median (IQR), mg/dL	*p*	Univariate Regression, OR (95%CI)	*p*	Multivariate Regression C, OR (95%CI)	*p*	AUC
***n***	128	162		290		290		290
**SM C16:0**	105 (83, 142)	109 (83, 136)	0.92	0.85 (0.23, 3.18)	0.81	1.46 (0.28, 7.51)	0.65	0.50
**SM C16:1**	14.75 (11.20, 20.45)	15.25 (11.20, 19.40)	0.82	0.78 (0.21, 2.89)	0.71	1.34 (0.25, 7.15)	0.74	0.51
**SM C18:0**	21.55 (17.4, 30.35)	21.35 (15.20, 29.70)	0.45	0.60 (0.18, 1.98)	0.40	1.27 (0.29, 5.63)	0.75	0.53
**SM C18:1**	10.35 (7.72, 13.15)	9.33 (6.96, 12.80)	0.32	0.55 (0.17, 1.78)	0.32	1.12 (0.25, 4.97)	0.88	0.53
**SM C20:2**	0.58 (0.35, 0.89)	0.61 (0.38, 0.85)	0.60	1.22 (0.46, 3.24)	0.68	1.50 (0.44, 5.10)	0.52	0.51
**SM C22:3**	3.26 (2.42, 5.35)	3.24 (2.06, 4.5)	0.13	0.49 (0.20, 1.24)	0.13	0.89 (0.29, 2.73)	0.84	0.55
**SM C24:0**	14.75 (10.6, 19.5)	12.65 (9.38, 16.7)	0.03	0.26 (0.08, 0.86)	0.03	0.43 (0.10, 1.90)	0.27	0.58
**SM C24:1**	34.60 (29.00, 46.55)	36.30 (27.80, 44.50)	0.91	0.82 (0.23, 2.90)	0.76	1.52 (0.31, 7.32)	0.60	0.50
**SM C26:0**	0.15 (0.13, 0.21)	0.15 (0.11, 0.23)	0.52	0.63 (0.25, 1.62)	0.34	0.78 (0.24, 2.47)	0.67	0.52
**SM C26:1**	0.27 (0.21, 0.37)	0.28 (.21, 0.36)	0.68	0.53 (0.18, 1.57)	0.25	0.67 (0.18, 2.57)	0.56	0.51
**SM (OH) C14:1**	6.51 (4.87, 8.68)	6.78 (4.41, 9.07)	0.77	0.71 (0.23, 2.18)	0.55	0.68 (0.17, 2.80)	0.59	0.51
**SM (OH) C16:1**	3.50 (2.72, 4.58)	3.59 (2.23, 5.04)	0.49	0.57 (0.19, 1.71)	0.32	0.68 (0.17, 2.70)	0.59	0.52
**SM (OH) C22:1**	9.55 (7.25, 12.6)	8.15 (5.75, 11.5)	0.01	0.20 (0.06, 0.66)	0.01	0.24 (0.05, 1.02)	0.05	0.59
**SM (OH) C22:2**	8.32 (6.16, 11.00)	7.63 (5.64, 10.1)	0.12	0.35 (0.10, 1.15)	0.08	0.30 (0.06, 1.37)	0.12	0.55
**SM (OH) C24:1**	1.08 (0.86, 1.32)	0.94 (0.71, 1.30)	0.01	0.19 (0.05, 0.67)	0.01	0.18 (0.04, 0.87)	0.03	0.59
**Total sum of SM**	237.80 (185.60, 309.60)	238.80 (174.40, 301.20)	0.63	0.61 (0.16, 2.33)	0.47	1.12 (0.21, 5.99)	0.90	0.52

Data are presented as number (*n*) and percentage (%) or median (interquartile range) or Odds Ratio (OR) and 95% Confidence Interval (CI). SM: Sphingomyelin; AUC: Area under the curve. Multivariate regression C: adjusted for age and gender and clinical shock markers.

**Table 3:** Spearman rank correlations of sphingomyelin species and clinical markers.

Spearman Rank Test		Heart Rate	Respiratory Rate	BP Systolic	BP Diastolic	No-Flow Time	Low-Flow Time	Troponin	Creatinine	Urea	Lactate	pH
**Total sum of SM**	rho	0.004	0.07	0.08	0.16	−0.04	−0.08	0.00	−0.08	−0.06	−0.02	0.11
	*p*-value	0.95	0.24	0.17	0.01	0.52	0.18	0.94	0.18	0.29	0.71	0.06
**SM C16:0**	rho	0.002	0.06	0.06	0.13	−0.01	−0.08	−0.02	−0.05	−0.03	−0.01	0.09
	*p*-value	0.97	0.30	0.31	0.03	0.84	0.18	0.72	0.40	0.61	0.92	0.11
**SM C16:1**	rho	−0.01	0.08	0.10	0.13	−0.04	−0.12	−0.01	−0.04	0.00	−0.04	0.16
	*p*-value	0.83	0.20	0.09	0.03	0.53	0.04	0.89	0.49	0.98	0.51	0.01
**SM C18:1**	rho	−0.01	0.08	0.14	0.17	−0.07	−0.10	−0.01	−0.10	−0.04	−0.04	0.15
	*p*-value	0.81	0.18	0.02	0.005	0.22	0.08	0.85	0.10	0.51	0.55	0.01
**SM C20:2**	rho	0.02	0.04	0.11	0.16	−0.04	−0.07	0.00	−0.11	−0.07	0.04	0.11
	*p*-value	0.74	0.53	0.07	0.01	0.52	0.21	1.00	0.06	0.26	0.47	0.07
**SM C22:3**	rho	−0.06	0.06	0.12	0.14	−0.08	−0.08	0.05	−0.08	−0.10	−0.07	0.16
	*p*-value	0.33	0.32	0.05	0.02	0.16	0.19	0.36	0.16	0.10	0.24	0.01
**SM C24:0**	rho	0.003	0.05	0.06	0.21	−0.06	0.02	0.15	−0.12	−0.19	−0.04	0.08
	*p*-value	0.96	0.37	0.34	0.01	0.34	0.76	0.01	0.04	0.002	0.54	0.19
**SM (OH) C14:1**	rho	−0.02	0.09	0.11	0.12	−0.11	−0.10	−0.01	−0.02	0.08	−0.03	0.08
	*p*-value	0.72	0.11	0.07	0.04	0.05	0.08	0.82	0.75	0.18	0.57	0.18
**SM (OH) C16:1**	rho	−0.01	0.09	0.09	0.14	−0.12	−0.09	−0.01	−0.03	0.05	−0.03	0.09
	*p*-value	0.81	0.14	0.12	0.02	0.04	0.13	0.85	0.58	0.42	0.63	0.12
**SM (OH) C22:1**	rho	−0.001	0.09	0.08	0.18	−0.15	−0.03	0.12	−0.09	−0.07	−0.03	0.07
	*p*-value	0.99	0.13	0.17	0.002	0.01	0.61	0.05	0.12	0.22	0.63	0.25
**SM (OH) C22:2**	rho	−0.03	0.10	0.09	0.14	−0.14	−0.07	0.03	−0.07	0.01	−0.03	0.07
	*p*-value	0.60	0.09	0.14	0.02	0.02	0.21	0.58	0.25	0.92	0.62	0.21
**SM (OH) C24:1**	rho	−0.03	0.07	0.04	0.15	−0.10	−0.01	0.11	−0.10	−0.09	−0.03	0.08
	*p*-value	0.67	0.22	0.50	0.01	0.09	0.91	0.06	0.09	0.11	0.65	0.15

Data is presented as correlation coefficient rho. SM: Sphingomyelin; BP: Blood pressure.

## Appendix A

**Table A1:** Univariate and multivariate analysis for in-hospital mortality.

	Survivors, Median (IQR), mg/dL	Non-Survivors, Median (IQR), mg/dL	*p*	Univariate Regression, OR (95%CI)	*p*	Multivariate Regression C. OR (95%CI)	*p*	AUC
***n***	161	129		290		290		290
**SM C16:0**	104 (82, 143)	111 (85.90, 136)	0.64	0.95 (0.26, 3.55)	0.94	1.30 (0.28, 5.97)	0.74	0.52
**SM C16:1**	14.60 (11.30, 20.00)	15.70 (11.20, 19.40)	0.92	0.86 (0.23, 3.18)	0.82	1.00 (0.21, 4.75)	1.00	0.50
**SM C18:0**	21.20 (16.90, 30.30)	21.70 (15.20, 29.30)	0.63	0.58 (0.18, 1.93)	0.38	0.86 (0.22, 3.47)	0.84	0.52
**SM C18:1**	10.10 (7.40, 13.00)	9.87 (7.01, 12.90)	0.59	0.60 (0.19, 1.94)	0.40	0.80 (0.20, 3.22)	0.76	0.52
**SM C20:2**	0.58 (0.35, 0.89)	0.61 (0.38, 0.85)	0.45	1.38 (0.52, 3.66)	0.51	1.43 (0.46, 4.50)	0.54	0.52
**SM C22:3**	3.24 (2.35, 5.18)	3.24 (2.06, 4.50)	0.22	0.49 (0.20, 1.24)	0.13	0.75 (0.26, 2.17)	0.60	0.55
**SM C24:0**	14.30 (10.20, 19.40)	12.80 (9.40, 16.60)	0.07	0.30 (0.10, 0.97)	0.05	0.48 (0.12, 1.88)	0.29	0.56
**SM C24:1**	35.10 (28.60, 46.20)	36.20 (27.80, 44.40)	0.79	0.67 (0.19, 2.37)	0.54	0.87 (0.20, 3.76)	0.86	0.51
**SM C26:0**	0.15 (0.12, 0.21)	0.16 (0.11, 0.23)	0.83	0.95 (0.37, 2.40)	0.91	1.25 (0.42, 3.69)	0.69	0.51
**SM C26:1**	0.28 (0.21, 0.37)	0.28 (0.21, 0.36)	0.45	0.50 (0.16, 1.53)	0.23	0.58 (0.15, 2.26)	0.44	0.53
**SM (OH) C14:1**	6.46 (4.80, 8.66)	6.88 (4.60, 9.07)	0.75	1.08 (0.35, 3.28)	0.89	0.97 (0.26, 3.62)	0.97	0.51
**SM (OH) C16:1**	3.4 (2.6, 4.57)	3.62 (2.23, 5.05)	0.84	0.73 (0.25, 2.17)	0.57	0.75 (0.21, 2.68)	0.66	0.51
**SM (OH) C22:1**	9.33 (6.90, 12.60)	8.19 (6.07, 11.50)	0.04	0.28 (0.09, 0.88)	0.03	0.34 (0.09, 1.28)	0.11	0.57
**SM (OH) C22:2**	8.18 (5.97, 10.70)	7.81 (5.91, 9.95)	0.33	0.49 (0.15, 1.59)	0.24	0.43 (0.11, 1.71)	0.23	0.53
**SM (OH) C24:1**	1.05 (.80, 1.33)	0.95 (0.71, 1.29)	0.05	0.28 (0.08, 0.96)	0.04	0.30 (0.07, 1.30)	0.11	0.57
**Total sum of SM**	233.20 (183.00, 309.50)	241.10 (182.60, 300.60)	0.90	0.67 (0.17, 2.54)	0.55	0.93 (0.20, 4.41)	0.93	0.50

Data are presented as number (*n*) and percentage (%) or median (interquartile range) or odds ratio (OR) and 95% confidence interval (CI). SM: Sphingomyelin; Multivariate regression C: adjusted for age, gender and clinical shock markers. AUC: Area under the curve.

**Table A2:** Multivariate analyses for modified Rankin Scale at hospital discharge.

	Multivariate Regression, Adjusted for Age and Gender. OR (95%CI)	*p*	Multivariate Regression, Adjusted for Age and Gender and Comorbidities. OR (95%CI)	*p*	Multivariate Regression, Adjusted for Age and Gender and Clinical Shock Markers. OR (95%CI)	*p*
***n***	290 (100%)		290 (100%)		290 (100%)	
**SM C16:0**	0.72 (0.18, 2.89)	0.65	0.94 (0.21, 4.30)	0.94	1.46 (0.28, 7.51)	0.65
**SM C16:1**	0.46 (0.11, 1.83)	0.27	0.74 (0.16, 3.40)	0.70	1.34 (0.25, 7.15)	0.74
**SM C18:0**	0.48 (0.14, 1.73)	0.26	0.69 (0.17, 2.83)	0.61	1.27 (0.29, 5.63)	0.75
**SM C18:1**	0.34 (0.10, 1.20)	0.09	0.57 (0.14, 2.27)	0.43	1.12 (0.25, 4.97)	0.88
**SM C20:2**	0.93 (0.34, 2.60)	0.90	1.33 (0.43, 4.12)	0.62	1.50 (0.44, 5.10)	0.52
**SM C22:3**	0.53 (0.20, 1.38)	0.19	0.70 (0.25, 1.97)	0.50	0.89 (0.29, 2.73)	0.84
**SM C24:0**	0.33 (0.10, 1.13)	0.07	0.58 (0.15, 2.26)	0.44	0.43 (0.10, 1.90)	0.27
**SM C24:1**	0.81 (0.22, 3.03)	0.76	1.15 (0.26, 5.13)	0.85	1.52 (0.31, 7.32)	0.60
**SM C26:0**	0.65 (0.24, 1.78)	0.41	0.95 (0.32, 2.83)	0.93	0.78 (0.24, 2.47)	0.67
**SM C26:1**	0.56 (0.17, 1.81)	0.34	0.66 (0.19, 2.36)	0.52	0.67 (0.18, 2.57)	0.56
**SM (OH) C14:1**	0.43 (0.13, 1.41)	0.16	0.65 (0.17, 2.40)	0.51	0.68 (0.17, 2.80)	0.59
**SM (OH) C16:1**	0.38 (0.12, 1.22)	0.10	0.57 (0.16, 2.07)	0.39	0.68 (0.17, 2.70)	0.59
**SM (OH) C22:1**	0.18 (0.05, 0.63)	0.01	0.34 (0.09, 1.34)	0.12	0.24 (0.05, 1.02)	0.05
**SM (OH) C22:2**	0.20 (0.06, 0.74)	0.02	0.37 (0.09, 1.53)	0.17	0.30 (0.06, 1.37)	0.12
**SM (OH) C24:1**	0.18 (0.05, 0.68)	0.01	0.30 (0.07, 1.27)	0.10	0.18 (0.04, 0.87)	0.03
**Total sum of SM**	0.50 (0.12, 2.05)	0.34	0.76 (0.16, 3.62)	0.73	1.12 (0.21, 5.99)	0.90

Data are presented as number (*n*) and percentage or odds ratio (OR) and 95% confidence interval (CI). SM: Sphingomyelin. Multivariate regression A: adjusted for age and gender. B: adjusted for age, gender, and comorbidities. C: adjusted for age, gender, and clinical shock markers.

**Table A3:** Multivariate analyses for in-hospital mortality.

	Multivariate Regression A. OR (95%CI)	*p*	Multivariate Regression B. OR (95%CI)	*p*	Multivariate Regression C. OR (95%CI)	*p*
***n***	290 (100%)		290 (100%)		290 (100%)	
**SM C16:0**	0.83 (0.21, 3.34)	0.80	0.79 (0.17, 3.62)	0.76	1.3 (0.28, 5.97)	0.74
**SM C16:1**	0.52 (0.13, 2.07)	0.35	0.61 (0.13, 2.8)	0.53	1 (0.21, 4.75)	0.99
**SM C18:0**	0.49 (0.14, 1.74)	0.27	0.52 (0.13, 2.12)	0.36	0.86 (0.22, 3.47)	0.84
**SM C18:1**	0.39 (0.11, 1.36)	0.14	0.51 (0.13, 2.02)	0.34	0.8 (0.2, 3.22)	0.76
**SM C20:2**	1.11 (0.4, 3.09)	0.84	1.32 (0.43, 4.02)	0.63	1.43 (0.46, 4.5)	0.54
**SM C22:3**	0.53 (0.21, 1.39)	0.20	0.65 (0.23, 1.84)	0.41	0.75 (0.26, 2.17)	0.6
**SM C24:0**	0.39 (0.12, 1.31)	0.13	0.54 (0.14, 2.14)	0.38	0.48 (0.12, 1.88)	0.29
**SM C24:1**	0.69 (0.18, 2.57)	0.58	0.8 (0.18, 3.5)	0.76	0.87 (0.2, 3.76)	0.86
**SM C26:0**	1.04 (0.38, 2.81)	0.94	1.24 (0.42, 3.67)	0.70	1.25 (0.42, 3.69)	0.69
**SM C26:1**	0.52 (0.15, 1.79)	0.30	0.58 (0.15, 2.24)	0.43	0.58 (0.15, 2.26)	0.44
**SM (OH) C14:1**	0.68 (0.21, 2.2)	0.52	0.75 (0.2, 2.73)	0.66	0.97 (0.26, 3.62)	0.97
**SM (OH) C16:1**	0.5 (0.16, 1.59)	0.24	0.55 (0.15, 1.98)	0.36	0.75 (0.21, 2.68)	0.66
**SM (OH) C22:1**	0.26 (0.08, 0.87)	0.03	0.34 (0.09, 1.34)	0.13	0.34 (0.09, 1.28)	0.11
**SM (OH) C22:2**	0.3 (0.09, 1.07)	0.06	0.4 (0.1, 1.66)	0.21	0.43 (0.11, 1.71)	0.23
**SM (OH) C24:1**	0.27 (0.07, 0.99)	0.05	0.32 (0.07, 1.38)	0.13	0.3 (0.07, 1.3)	0.11
**Total sum of SM**	0.57 (0.14, 2.33)	0.43	0.63 (0.13, 2.99)	0.56	0.93 (0.2, 4.41)	0.93

Data are presented as number (n) and percentage (%) or odds ratio (OR) and 95% confidence interval (CI). SM: Sphingomyelin. Multivariate regression A: adjusted for age and gender. B: adjusted for age, gender, and comorbidities. C: adjusted for age, gender, and clinical shock markers.

**Table A4:** Univariate analysis for all-cause mortality after 1 year.

	Survivors, Median (IQR), mg/dL	Non-Survivors, Median (IQR), mg/dL	*p*	Univariate Regression, OR (95%CI)	*p*	AUC
***n***	120 (41.4%)	149 (51.4%)		269 (92.8%)		269 (92.8%)
**SM C16:0**	104 (81.95, 144.50)	110 (87, 136)	0.56	1.19 (0.31, 4.63)	0.80	0.52
**SM C16:1**	14.65 (11.35, 20)	15.70 (11.20, 19.50)	0.83	1.10 (0.28, 4.22)	0.89	0.51
**SM C18:0**	21.65 (17.05, 31.75)	21.70 (15.9, 29.1)	0.54	0.61 (0.18, 2.07)	0.42	0.52
**SM C18:1**	10.15 (7.465, 13.25)	9.99 (7.01, 12.8)	0.47	0.61 (0.18, 2.02)	0.42	0.53
**SM C20:2**	0.58 (0.36, 0.89)	0.62 (0.38, 0.87)	0.57	1.25 (0.46, 3.44)	0.66	0.52
**SM C22:3**	3.26 (2.23, 5.42)	3.29 (2.15, 4.5)	0.27	0.57 (0.23, 1.46)	0.24	0.54
**SM C24:0**	14.50 (10.35, 19.8)	12.80 (9.7, 16.6)	0.06	0.24 (0.07, 0.86)	0.03	0.57
**SM C24:1**	34.40 (28.6, 46.55)	36.50 (28.9, 45.1)	0.70	0.96 (0.26, 3.59)	0.96	0.51
**SM C26:0**	0.15 (0.12, 0.22)	0.15 (0.11, 0.23)	0.80	0.68 (0.26, 1.8)	0.44	0.51
**SM C26:1**	0.28 (0.21, 0.37)	0.29 (0.21, 0.36)	0.53	0.46 (0.14, 1.44)	0.18	0.52
**SM (OH) C14:1**	6.48 (4.82, 8.68)	6.88 (4.6, 9.07)	0.95	0.92 (0.29, 2.93)	0.89	0.50
**SM (OH) C16:1**	3.43 (2.66, 4.65)	3.62 (2.33, 5.04)	0.6	0.58 (0.19, 1.81)	0.35	0.52
**SM (OH) C22:1**	9.59 (7.20, 12.9)	8.19 (6.07, 11.3)	0.01	0.18 (0.05, 0.61)	0.01	0.59
**SM (OH) C22:2**	8.32 (6.09, 10.95)	7.77 (5.5, 10.1)	0.16	0.36 (0.1, 1.25)	0.11	0.55
**SM (OH) C24:1**	1.07 (0.86, 1.43)	0.97 (0.72, 1.29)	0.02	0.18 (0.05, 0.69)	0.01	0.58
**Total sum of SM**	235.5 (180.9, 313.2)	241.10 (186.80, 300.60)	0.97	0.78 (0.2, 3.09)	0.72	0.50

Data are presented as number (n) and percentage (%) or median (interquartile range) or odds ratio (OR) and 95% confidence interval (CI). SM: Sphingomyelin; AUC: Area under the curve.

**Table A5:** Multivariate analyses for all-cause mortality after 1 year.

	Multivariate Regression A. OR (95%CI)	*p*	Multivariate Regression B. OR (95%CI)	*p*	Multivariate Regression C. OR (95%CI)	*p*
***n***	269 (92.8)		269 (92.8)		269 (92.8)	
**SM C16:0**	1.00 (0.23, 4.27)	1.00	1.31 (0.26, 6.67)	0.74	1.72 (0.34, 8.81)	0.52
**SM C16:1**	0.59 (0.14, 2.54)	0.48	0.90 (0.18, 4.58)	0.90	1.32 (0.25, 7.12)	0.74
**SM C18:0**	0.48 (0.13, 1.8)	0.28	0.70(0.16, 3.07)	0.63	0.95 (0.22, 4.19)	0.95
**SM C18:1**	0.36 (0.1, 1.33)	0.13	0.59 (0.14, 2.52)	0.47	0.82 (0.19, 3.63)	0.80
**SM C20:2**	0.97 (0.33, 2.84)	0.95	1.41 (0.42, 4.7)	0.58	1.35 (0.39, 4.67)	0.63
**SM C22:3**	0.63 (0.23, 1.68)	0.35	0.77 (0.26, 2.25)	0.63	0.97 (0.32, 2.91)	0.95
**SM C24:0**	0.33 (0.09, 1.24)	0.1	0.67 (0.15, 2.97)	0.60	0.50 (0.11, 2.17)	0.35
**SM C24:1**	1.03 (0.25, 4.18)	0.97	1.46 (0.29, 7.42)	0.65	1.84 (0.38, 8.81)	0.45
**SM C26:0**	0.73 (0.26, 2.1)	0.56	1.11 (0.35, 3.46)	0.86	0.90 (0.28, 2.88)	0.86
**SM C26:1**	0.47 (0.13, 1.71)	0.25	0.57 (0.14, 2.38)	0.44	0.60 (0.15, 2.44)	0.47
**SM (OH) C14:1**	0.49 (0.14, 1.73)	0.27	0.69 (0.17, 2.78)	0.6	0.66 (0.16, 2.74)	0.57
**SM (OH) C16:1**	0.34 (0.1, 1.18)	0.09	0.46 (0.12, 1.85)	0.28	0.50 (0.12, 2.03)	0.33
**SM (OH) C22:1**	0.15 (0.04, 0.57)	0.01	0.29 (0.07, 1.27)	0.10	0.21 (0.05, 0.94)	0.04
**SM (OH) C22:2**	0.19 (0.05, 0.76)	0.02	0.33 (0.07, 1.54)	0.16	0.28 (0.06, 1.3)	0.10
**SM (OH) C24:1**	0.16 (0.04, 0.69)	0.01	0.29 (0.06, 1.44)	0.13	0.22 (0.04, 1.08)	0.06
**Total sum of SM**	0.63 (0.14, 2.77)	0.54	0.96 (0.18, 5.1)	0.97	1.19 (0.22, 6.34)	0.84

Data are presented as number (*n*) or odds ratio (OR), 95% confidence interval (CI). SM: Sphingomyelin. Multivariate regression A: adjusted for age and gender. B: adjusted for age, gender, and comorbidities. C: adjusted for age, gender, and clinical shock markers.
